# An ultrasensitive reverse transcription polymerase chain reaction assay to detect asymptomatic low-density *Plasmodium falciparum* and *Plasmodium vivax* infections in small volume blood samples

**DOI:** 10.1186/s12936-015-1038-z

**Published:** 2015-12-23

**Authors:** Matthew Adams, Sudhaunshu N. Joshi, Gillian Mbambo, Amy Z. Mu, Shay M. Roemmich, Biraj Shrestha, Kathy A. Strauss, Nicole Eddington Johnson, Khine Zaw Oo, Tin Maung Hlaing, Zay Yar Han, Kay Thwe Han, Si Thura, Adam K. Richards, Fang Huang, Myaing M. Nyunt, Christopher V. Plowe

**Affiliations:** Howard Hughes Medical Institute/Institute for Global Health, University of Maryland School of Medicine, Baltimore, USA; Defence Services Medical Research Centre, Ministry of Defence, Nay Pyi Taw, Myanmar; Department of Medical Research, Ministry of Health, Yangon, Myanmar; Community Partners International, Yangon, Myanmar; UCLA Division of General Internal Medicine and Health Services Research, Los Angeles, USA; National Institute of Parasitic Diseases, Chinese Center for Disease Control and Prevention, Shanghai, People’s Republic of China

**Keywords:** Malaria, Malaria elimination, *Plasmodium falciparum*, *Plasmodium vivax*, RT-PCR, Limits of detection, Nucleic acid

## Abstract

**Background:**

Highly sensitive, scalable diagnostic methods are needed to guide malaria elimination interventions. While traditional microscopy and rapid diagnostic tests (RDTs) are suitable for the diagnosis of symptomatic malaria infection, more sensitive tests are needed to screen for low-density, asymptomatic infections that are targeted by interventions aiming to eliminate the entire reservoir of malaria infection in humans.

**Methods:**

A reverse transcription polymerase chain reaction (RT- PCR) was developed for multiplexed detection of the 18S ribosomal RNA gene and ribosomal RNA of *Plasmodium falciparum* and *Plasmodium vivax*. Simulated field samples stored for 14 days with sample preservation buffer were used to assess the analytical sensitivity and specificity. Additionally, 1750 field samples from Southeastern Myanmar were tested both by RDT and ultrasensitive RT-PCR.

**Results:**

Limits of detection (LoD) were determined under simulated field conditions. When 0.3 mL blood samples were stored for 14 days at 28 °C and 80 % humidity, the LoD was less than 16 parasites/mL for *P. falciparum* and 19.7 copies/µL for *P. vivax* (using a plasmid surrogate), about 10,000-fold lower than RDTs. Of the 1739 samples successfully evaluated by both ultrasensitive RT-PCR and RDT, only two were RDT positive while 24 were positive for *P. falciparum*, 108 were positive for *P. vivax*, and 127 were positive for either *P. vivax* and/or *P. falciparum* using ultrasensitive RT-PCR.

**Conclusions:**

This ultrasensitive RT-PCR method is a robust, field-tested screening method that is vastly more sensitive than RDTs. Further optimization may result in a truly scalable tool suitable for widespread surveillance of low-level asymptomatic *P. falciparum* and *P. vivax* parasitaemia.

**Electronic supplementary material:**

The online version of this article (doi:10.1186/s12936-015-1038-z) contains supplementary material, which is available to authorized users.

## Background

Momentum is growing for a campaign to eliminate malaria from Southeast Asia in response to the emergence there of multidrug-resistant *Plasmodium falciparum* [1]. In pilot studies of a possible elimination strategy, high-volume, quantitative polymerase chain reaction (qPCR) is being used to identify villages along the Thailand-Myanmar border whose inhabitants have a high prevalence of sub-microscopic, asymptomatic *P. falciparum* infections [2]. These ultrasensitive tests reveal much higher prevalences of infection than those detected by less sensitive standard microscopy, rapid diagnostic tests (RDTs) or polymerase chain reaction (PCR) using Deoxyribonucleic Acid (DNA) extracted from filter paper samples. The prevalence rates detected by high-volume qPCR are being used to select populations for targeted mass drug treatment to eliminate malaria. Current methods are unscalable however, as they require the collection of 1–2 mL of venous blood, centrifugation and buffy coat removal, and freezing of samples in liquid nitrogen [2] or other cold transport [3] to achieve ultrasensitive detection. Additionally, these methods are genus-specific, and do not differentiate between *P. falciparum* and the other common malaria species in Asia, *Plasmodium vivax* in a single test.

Current high-volume qPCR methods target the DNA of the *P. falciparum* multi-copy 18S ribosomal RNA (18SrRNA) gene which has five copies in every parasite genome (chromosomes 1, 5, 7, 11 and 13), thus lowering the limit of detection about fivefold compared to molecular tests targeting single copy genes. However, a single asexual parasite circulating in peripheral blood has roughly 3500 18SrRNA transcripts [4], providing thousands-fold more potential targets in a given blood volume. Until now, PCR-based detection methods for malaria surveillance typically targeted only DNA because ribonucleic acid (RNA) is thought to be too unstable to withstand sample storage, transport and processing under field conditions.

An improved assay was developed to detect both ribosomal RNA and DNA, thus taking advantage of the high copy numbers of RNA in small blood volumes, using a stabilizing reagent that allows preservation of nucleic acids from small volumes of blood suitable for collection by finger or ear stick, with minimal sample processing, and no cold chain.

## Methods

### Stabilization, extraction of nucleic acids and reverse transcription-PCR

A 300 µL volume of patient blood was mixed with 750 µL of DNA/RNA Shield (Zymo Research, Irvine, CA, USA), stored up to 2 weeks at 28 °C or below, then frozen at −80 °C until extraction. Total nucleic acid was extracted from 200 μL of blood/stabilizer mixture using the QIAamp 96 DNA Blood Kit (Qiagen, Valencia, CA, USA) following the manufacturer’s instructions, but with an elution volume of 50 µL of Buffer AE. RT-PCR was performed with modifications as previously described [5, 6]. Specifically, a 10 µL reaction containing 5 µL 2 × Quantitect Multiplex Master Mix, 0.1 µL Quantitect Reverse Transcriptase Mix (Qiagen, Valencia, CA, USA), 0.2 µL 1U/µL heat-labile uracil-DNA glycosolase (Roche Diagnostics, Indianapolis, IN, USA), 1.5 µL nucleic acid template, RNAse-free water and the primers/probes listed in Additional file 1, was used to detect parasite nucleic acid. The *P. vivax* and *P. falciparum* primers and probes were adapted with a modified fluorophore [5] and combined with the same internal control primers and probes as the recently published described for high-volume qPCR [2]. RT-PCR was performed on a Roche Lightcycler 96 or 480 real time PCR machine (Roche Diagnostics, Indianapolis, IN, USA) following the cycling conditions in the Additional file 1. Results were analysed with the LC96 software version 1.1 or the LC480 software 1.5.1. Quantitation cycle (Cq) values were determined by the proprietary algorithm on the LC96 software and using the ‘fit points’ method with the LC480.

### *Plasmodium falciparum* limits of detection under simulated field storage conditions

To measure the limits of detection (LoD) for *P. falciparum*, mock samples were created to simulate field infections. Synchronous, ring-state NF54 parasites were obtained by a combination of sorbitol synchronization and magnetic separation using the QuadroMACS separator system with LD columns (Miltenyi Biotec, Auburn, CA, USA). Parasites were quantified by microscopy then serially diluted in parasite culture media followed by a final dilution in malaria-naïve human whole blood, resulting in final parasite concentrations of 250,000, 50,000, 10,000, 2000, 400, 80, 16 and 0 parasites/mL (p/mL).

Three independent experiments were performed to measure the baseline LoD of *P. falciparum* under ambient storage conditions (3 days at 22 °C). Each experiment followed the standard curve design above and was performed with eight replicates per dilution. The LoD was determined using a probit analysis in SAS 9.2 (SAS Institute, Cary, NC, USA).

The impact of tropical storage conditions after sample collection on the *P. falciparum* LoD was also evaluated. The simulated samples, each prepared in quadruplicate, were frozen at −80 °C or stored for three, seven or 14 days at 28 °C with 80 % relative humidity (RH) prior to extraction. LoD was calculated by probit as above.

### Limits of detection in admixtures of *Plasmodium falciparum* and *Plasmodium vivax*

The LoD for *P. falciparum* was determined by preparing and testing three independent sets of *P. falciparum* standard curves as described above, both in the presence and absence of 3.8 × 10^4^ copies/μL of PUC57 plasmid containing a *P. vivax* 18srRNA gene insert (obtained from Gary Fahle, National Institutes of Health Vaccine Research Center). Since *P. vivax* parasites are not sustainable in culture, this plasmid was used as a surrogate and reported in copies per μL. It should be noted that this plasmid surrogate will not represent the increased sensitivity due to RNA present in recently collected parasites. The LoD for *P. vivax* was determined by preparing and testing three independent sets of *P. vivax* plasmid standard curves (prepared by making serial 10-fold dilutions of the plasmid) resulting in concentrations ranging from 3.8 × 10^4^ to 3.8 × 10^−1^ copies/μL, both alone and in presence of total nucleic acid from 1 × 10^4^ p/mL of NF54 parasites in blood. Each standard dilution was tested in triplicate.

Negative predictive value (NPV), positive predictive value (PPV), analytical sensitivity, and analytical specificity were calculated using SAS 9.2 (SAS Institute, Cary, NC, USA) along with the 95 % confidence intervals using a two-sided Fisher’s exact test. A McNemar’s test was performed using Graphpad. From the probit analysis, a LoD was determined in terms of parasites per millilitre for *P. falciparum* or plasmid copies per μL for *P. vivax*. Samples below the determined LoD were not expected to be positive, and were omitted from these analyses.

### Field test of samples from Southeastern Myanmar

As part of a cross-sectional survey, samples were collected across three townships in Southeastern Myanmar following a protocol approved by the Ethics Review Board of the Department of Medical Research of the Myanmar Ministry of Health. The detailed results of this survey will be described in a separate presentation. Blood collected by finger stick was used to perform RDTs (Malaria Ag P.f/Pv, Standard Diagnostics, Republic of Korea) on site, and 0.3 mL of blood was placed into collection tubes as described above. Anticoagulated blood was mixed with the stabilizer and then transported at ambient temperature to a central laboratory in Yangon for storage at −80 °C within 10 days of collection. The frozen samples were then shipped to Baltimore where extraction and ultrasensitive RT-PCR detection were performed in replicate as described above.

## Results

A baseline LoD under ambient storage conditions was established by performing three independent experiments with eight replicates of each parasite concentration per experiment. All 24 replicates of *P. falciparum* culture material diluted in uninfected human whole blood, ranging from 250,000 to 80 p/mL, were detected as positive when stored for 3 days at 22 °C. Of the 24 replicates at 16 p/mL, 18 were detected as positive. The LoD under these conditions was determined to be 20.5 p/mL.

The impact of sample storage at higher temperatures and RH was evaluated in quadruplicate. All four replicates for each concentration of parasites (250,000 to 80 p/mL) were detected as positive when samples were immediately frozen at −80 °C or stored for three, seven or 14 days at 28 °C in 80 % RH before processing. The 16 p/mL concentration was detected in two of four replicates when samples were frozen, as compared to three of four, four of four and four of four when samples were stored for three, seven or 14 days, respectively. The LoD of *P. falciparum* samples when frozen at −80 °C was 23.3 p/mL, and when stored for three, seven or 14 days in tropical conditions was 20.5, <16 and <16 p/mL, respectively.

To assess the effect of admixtures of *P. falciparum* with *P. vivax*, nucleic acid from cultured *P. falciparum* parasites diluted in human blood was assessed with and without the addition of a *P. vivax* plasmid surrogate. All nine replicates of each *P. falciparum* concentration from 250,000 to 80 p/mL were detected in the presence and absence of 3.8 × 10^4^ copies/μL of *P. vivax* plasmid in the RT-PCR template. Of the nine replicates of 16 p/mL *P. falciparum*, six were detected when not mixed and three were detected when mixed. The resulting LoD of *P. falciparum* when mixed with *P. vivax* was 25.1 p/mL, and when not mixed was 21.3 p/mL.

The LoD of *P. vivax* plasmid with and without added *P. falciparum* (1 × 10^4^ p/mL) and human nucleic acid was evaluated. All nine replicates were detected for each plasmid concentration, from 5.7 × 10^4^ copies/μL to 5.7 × 10^2^ copies/μL. In the absence of *P. falciparum*/human nucleic acid, the plasmid was detected nine of nine times at 5.7 × 10^1^ copies/μL, six of nine at 5.7 × 10^0^ copies/μL, and two of nine at 5.7 × 10^−1^ copies/μL. The plasmid, when combined with *P. falciparum*/human nucleic acid, was detected seven of nine times at 5.7 × 10^1^ copies/μL, zero of nine at 5.7 × 10^0^ copies/μL, and zero of nine at 5.7 × 10^−1^ copies/μL. The LoD of the plasmid was 19.7 copies/μL. In the presence of ‘contaminating’ *P. falciparum*/human nucleic acid, the LoD of the plasmid increased to 49.5 copies/μL.

The LoD for each species as well as the impact of varied storage conditions on the LoD are shown in Table 1. Sensitivity, specificity, positive predictive value, and negative predictive are shown in Table 2.

**Table 1:** Limits of detection of *Plasmodium falciparum* under varied storage conditions of *Plasmodium falciparum* and *Plasmodium vivax* admixtures

	Sample collection storage conditions				Admixture of *P. falciparum* and *P. vivax*			
	3 days at 22 °C	Frozen (−80 °C)	3 days at 28 °C 80 % RH	7 or 14 days at 28 °C 80 % RH	Pf only	Pv only	Pf w/Pv plasmid^a^	Pv plasmid w/Pf^b^
Pf LoD (p/mL)	20.5	23.3	20.5	<16	21.3	–	25.1	–
Pv LoD (copies/μL)	–	–	–	–	–	19.7	–	49.5

*LoD* Limits of detection; *Pf* *P. falciparum*; *Pv* *P. vivax*; *RH* relative humidity

^a^3.8 × 10^4^ plasmid copies/μL

^*b*^1 × 10^4^ p/mL *P. falciparum*

**Table 2:** Sensitivity, specificity, positive predictive value and negative predictive value of the RT-PCR performed on simulated samples

Species test	*P. falciparum*		*P. vivax*	
	Positive	Negative	Positive	Negative
Species-specific RT-PCR				
Positive	522	0	154	0
Negative	2	451	2	876

McNemar’s (two-tailed) test	P = 0.4795			
	Percentage	95 % CI	Percentage	95 % CI
Sensitivity	100	99.30–100	100	97.66–100
Specificity	99.56	98.41–99.95	99.77	99.18–99.97
PPV	99.62	98.63–99.95	98.73	95.50–99.85
NPV	100	99.19–100	100	99.58–100

The analysis of field samples showed a large proportion of infections that were undetectable by RDT. Only two samples were positive for *P. falciparum* by RDT. Ultrasensitive RT-PCR detected 24 *P. falciparum* positives and 108 *P. vivax* positives with 127 infections being either *P. falciparum* and/or *P. vivax*. Although the number of positives detected by RDT was small, both of the RDT-positive infections were also positive by ultrasensitive RT-PCR.

A small fraction of field samples were positive in one extracted well but not the other. These samples with incongruous results were retested via RT-PCR in quadruplicate. Of the two non-replicating *P. falciparum* positive samples, one was detected in all four replicates and the other was repeatedly negative. Of the six non-replicating *P. vivax* positive samples, three were repeatedly positive, while one was negative. The remaining two samples were of mixed results suggesting a very low-density infection near the LoD.

## Discussion

This assay represents an extremely sensitive, robust and potentially scalable procedure for collecting small volume blood specimens under field conditions to conduct molecular surveillance for *P. falciparum* and *P. vivax*. Building on previous work [2, 5, 6], these improved methods for stabilizing nucleic acids before processing and detecting both DNA and RNA will permit estimation of malaria prevalence even in hard-to-reach areas far from refrigeration or laboratory facilities.

This procedure currently uses 0.3 mL of blood collected from a finger or ear stick. While this volume is routinely collected in field studies, to be truly scalable the sample volume should be closer to the approximately 50 μL of finger stick blood typically collected by peripheral health care workers for RDTs. A larger volume was used to ensure that at least some parasite material would be present in the collected blood. However, the nucleic acid stabilizer may allow for smaller volumes of blood to be collected by disrupting infected red blood cells and dispersing thousands of copies of nucleic acid per parasite throughout the blood/stabilizer mixture. Since only approximately 57 μL of blood is used for nucleic acid extraction, additional improvements can be made to decrease the volume of blood drawn while maintaining sensitivity.

Although intrinsically the RT-PCR reporter assay is more sensitive than reported, limiting PCR cycles to as few as 37 allows for a LoD of 25 parasites/mL while minimizing the risk of false positives. As with all highly sensitive detection methods, a strict protocol to prevent false positives from cross-contamination is required. In earlier versions of this assay, each instance of a false positive was investigated by root cause analysis which identified cross-contamination from adjacent highly parasitaemic samples or positive controls. This contamination was subsequently minimized by adhering to a strict protocol including the use of mechanical barriers during extraction and RT-PCR setup, using several negative controls, and limiting the number of cycles used for analysis.

## Conclusion

Ongoing malaria surveys using 0.3 mL samples in Myanmar and China will provide estimates of the prevalence of *P. falciparum* and *P. vivax* infection across a range of epidemiological settings. While the current assay will not provide a precise quantitation of parasites/mL in these field samples, it is possible to infer the relative prevalence of very low parasitaemia infections based on the RT-PCR threshold cycle. In other words, with clinical malaria representing the ‘tip of the iceberg’, use of an ultra-sensitive detection method will reveal the shape of the submerged iceberg, i.e., the distribution of sub-patent infections with relatively high, medium and low parasite densities. If very low density infections, those near the current LoD of the assay, represent only a small fraction of the asymptomatic reservoir of infection, then a compromise allowing for both a decrease in the volume of blood collected and an increase in the threshold for a positive test result, will further improve scalability and reduce the risk of false positive tests. If these refinements based on field testing are successful, and especially if ongoing efforts to achieve similar low LoDs using dried blood spots succeed, this ultrasensitive multiplexed malaria test has the potential to become part of a minimum standard toolkit for surveillance for malaria elimination campaigns in the Greater Mekong Subregion and elsewhere. Anticipating these improvements of the assay, capacity is being established for high throughput molecular surveillance for *P. falciparum* and *P. vivax* malaria in civilian and military laboratories in Myanmar, and technical support is available for malaria surveillance laboratories to perform this test worldwide.

## Additional file

10.1186/s12936-015-1038-z Primers, probes, cycling condition.

## References

[1] Plowe CV. The danger of untreatable malaria is real and present. Center for Strategic and International Studies. Washington DC: 2015.

[2] Imwong M, Hanchana S, Malleret B, Renia L, Day NP, Dondorp A (2014). High-throughput ultrasensitive molecular techniques for quantifying low-density malaria parasitemias. J Clin Microbiol.

[3] Canier L, Khim N, Kim S, Eam R, Khean C, Loch K (2015). Malaria PCR detection in cambodian low-transmission settings: dried blood spots versus venous blood samples. Am J Trop Med Hyg.

[4] Murphy SC, Prentice JL, Williamson K, Wallis CK, Fang FC, Fried M (2012). Real-time quantitative reverse transcription PCR for monitoring of blood-stage *Plasmodium falciparum* infections in malaria human challenge trials. Am J Trop Med Hyg.

[5] Kamau E, Tolbert LS, Kortepeter L, Pratt M, Nyakoe N, Muringo L (2011). Development of a highly sensitive genus-specific quantitative reverse transcriptase real-time PCR assay for detection and quantitation of plasmodium by amplifying RNA and DNA of the 18S rRNA genes. J Clin Microbiol.

[6] Seder RA, Chang LJ, Enama ME, Zephir KL, Sarwar UN, Gordon IJ (2013). Protection against malaria by intravenous immunization with a nonreplicating sporozoite vaccine. Science.

