# Supplementary material for: An ultrasensitive reverse transcription polymerase chain reaction assay to detect asymptomatic low-density Plasmodium falciparum and Plasmodium vivax infections in small volume blood samples
Source: Malar J. 2015 Dec 23;14:520. doi: 10.1186/s12936-015-1038-z (PMC4690410; doi:10.1186/s12936-015-1038-z)

Supplementary Table 1. Primers, probes, cycling conditions

Species	Primer or probe	[µM]	Sequence (5'- 3')[b]	Ref.	Cycling Conditions	
P. falciparum	Falciparum F	0.45	CCGACTAGGTGTTGGATGAAAGTGTTAA	(5)		
	Plasmodium R	0.60	AACCCAAAGACTTTGATTTCTCATAA		<u>RT-PCR Program</u>	<u>Ramp speed</u>
	Falciparum P	0.10	*Cy5*-AGCAATCTAAAAGTCACCTCGAAAGATGACT-*BHQ2*		50°C for 20 minutes (RT)	1.7°C/s
P. vivax	Vivax R	0.10	CCGACTAGGCTTTGGATGAAAGATTTTA		95°C 15 minutes	1.7°C/s
	Vivax P	0.10	*FAM*-AGCAATCTAAGAATAAACTCCGAAGAGAAAATTCT-*BHQ2*		94°C 45 seconds	1.7°C/s
Human	Actin F	0.10	ACCGAGCGCGGCTACAG	[2]	60°C 75 seconds (Data)	1.7°C/s
	Actin R	0.10	CTTAATGTCACGCACGATTTCC		Repeat to 94oC 44 times	
	Actin P	0.10	*VIC*-TTCACC ACCACGGCCGAGC-*MGB*			

[a]Probe sequence is as previously published [6], with modified fluorophores.

[b]Cy5, cyanine; BHQ_2, black hole quencher 2; FAM, carboxyfluorescein; VIC (Life Technologies, Carlsbad, CA); MGB, minor-groove binder

Supplementary Table 2. Resulting Ct values and statistical information showing impact of storage time on Ct values

	14 Days @ 28 ºC/80% RH			3 Days @ 28 ºC/80% RH		
parasites/mL	Cq	Cq Mean	Cq Error	Cq	Cq Mean	Cq Error
250,000	18.69	20.68	1.40	20.47	20.98	0.47
250,000	20.95	20.68	1.40	20.72	20.98	0.47
250,000	21.96	20.68	1.40	21.19	20.98	0.47
250,000	21.11	20.68	1.40	21.52	20.98	0.47
50,000	24.70	24.27	0.45	22.39	23.17	0.57
50,000	23.80	24.27	0.45	23.47	23.17	0.57
50,000	24.59	24.27	0.45	23.13	23.17	0.57
50,000	23.97	24.27	0.45	23.68	23.17	0.57
10,000	26.55	26.44	0.33	24.53	25.45	0.67
10,000	26.27	26.44	0.33	25.40	25.45	0.67
10,000	26.85	26.44	0.33	25.84	25.45	0.67
10,000	26.10	26.44	0.33	26.03	25.45	0.67
2,000	29.34	28.79	0.45	29.34	28.79	0.45
2,000	28.58	28.79	0.45	28.58	28.79	0.45
2,000	28.94	28.79	0.45	28.94	28.79	0.45
2,000	28.29	28.79	0.45	28.29	28.79	0.45
400	32.90	31.14	1.54	32.90	31.14	1.54
400	31.10	31.14	1.54	31.10	31.14	1.54
400	31.41	31.14	1.54	31.41	31.14	1.54
400	29.16	31.14	1.54	29.16	31.14	1.54
80	34.94	32.84	1.43	31.94	32.33	0.28
80	32.30	32.84	1.43	32.53	32.33	0.28
80	32.42	32.84	1.43	32.33	32.33	0.28
80	31.70	32.84	1.43	32.52	32.33	0.28
16	36.25	35.17	0.83	33.71	34.24	0.63
16	34.68	35.17	0.83	34.07	34.24	0.63
16	35.34	35.17	0.83	34.94	34.24	0.63
16	34.39	35.17	0.83	-	-	-
0	-	-	-	-	-	-
0	-	-	-	-	-	-
0	-	-	-	-	-	-
0	-	-	-	-	-	-

Supplementary Table 3. Evaluation of inter-assay and intra-assay variability and removal of reverse transcriptase from RT PCR on 250,000 *Plasmodium falciparum* parasites per mL in whole blood

	With Reverse Transcriptase			Without Reverse Transcriptase		
	**Cq**	**Cq Mean**	**Cq Error**	**Cq**	**Cq Mean**	**Cq Error**
Experiment 1	18.87	18.93	0.10	29.99	29.98	0.31
Experiment 1	19.02	18.93	0.10	29.53	29.98	0.31
Experiment 1	18.82	18.93	0.10	30.23	29.98	0.31
Experiment 1	19.02	18.93	0.10	30.15	29.98	0.31
Experiment 2	18.61	18.69	0.20			
Experiment 2	18.95	18.69	0.20			
Experiment 2	18.47	18.69	0.20			
Experiment 2	18.74	18.69	0.20			
Experiment 3	18.81	18.85	0.11			
Experiment 3	18.99	18.85	0.11			
Experiment 3	18.74	18.85	0.11			
Experiment 3	18.86	18.85	0.11			

Supplementary Figure 1. Example data output from standard curve of serially diluted *P. falciparum* parasites diluted in whole blood

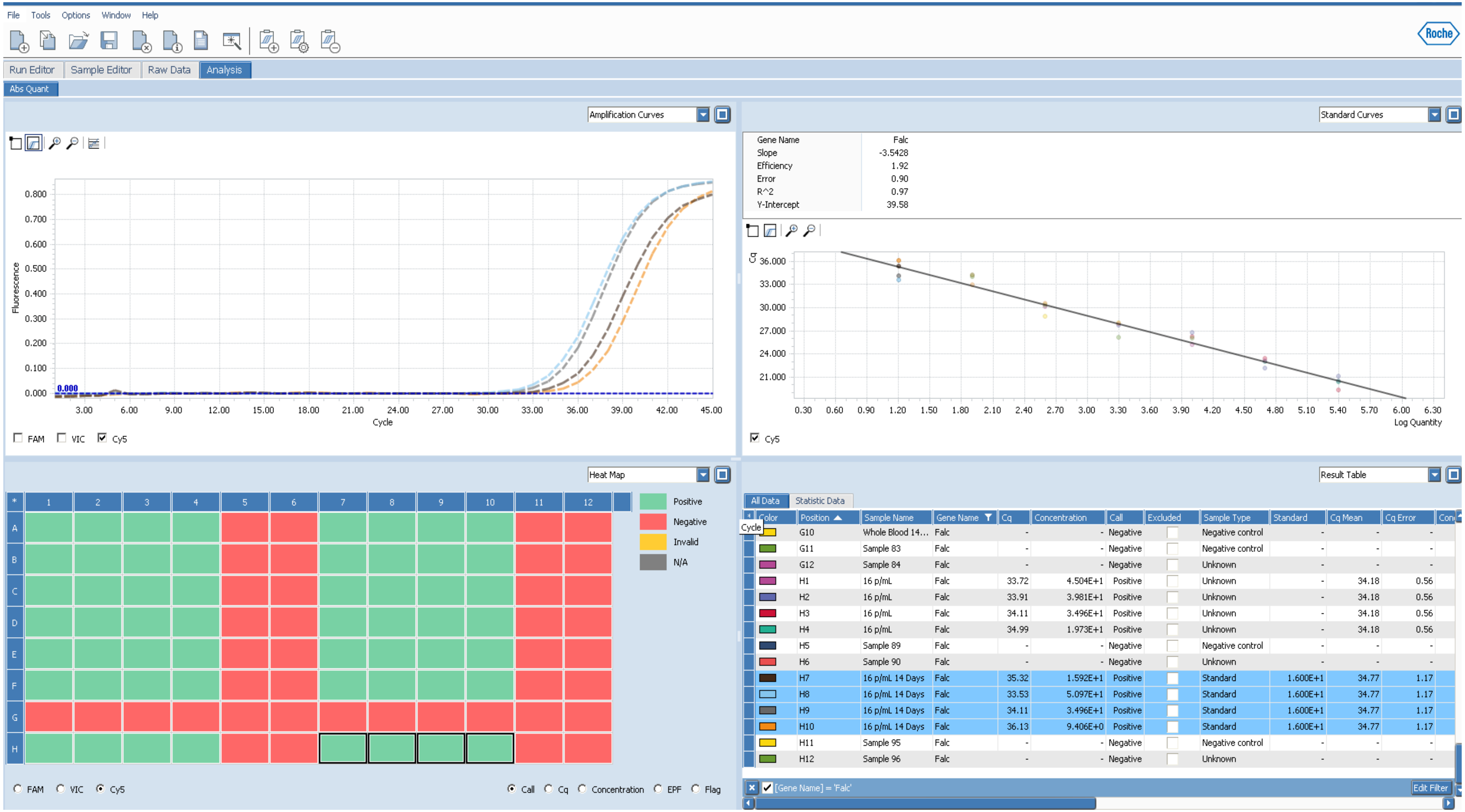

Supplementary Figure 2. Example data output from standard curve of serially diluted *P. vivax* plasmid diluted in 10^4 *P. falciparum* parasites and whole blood

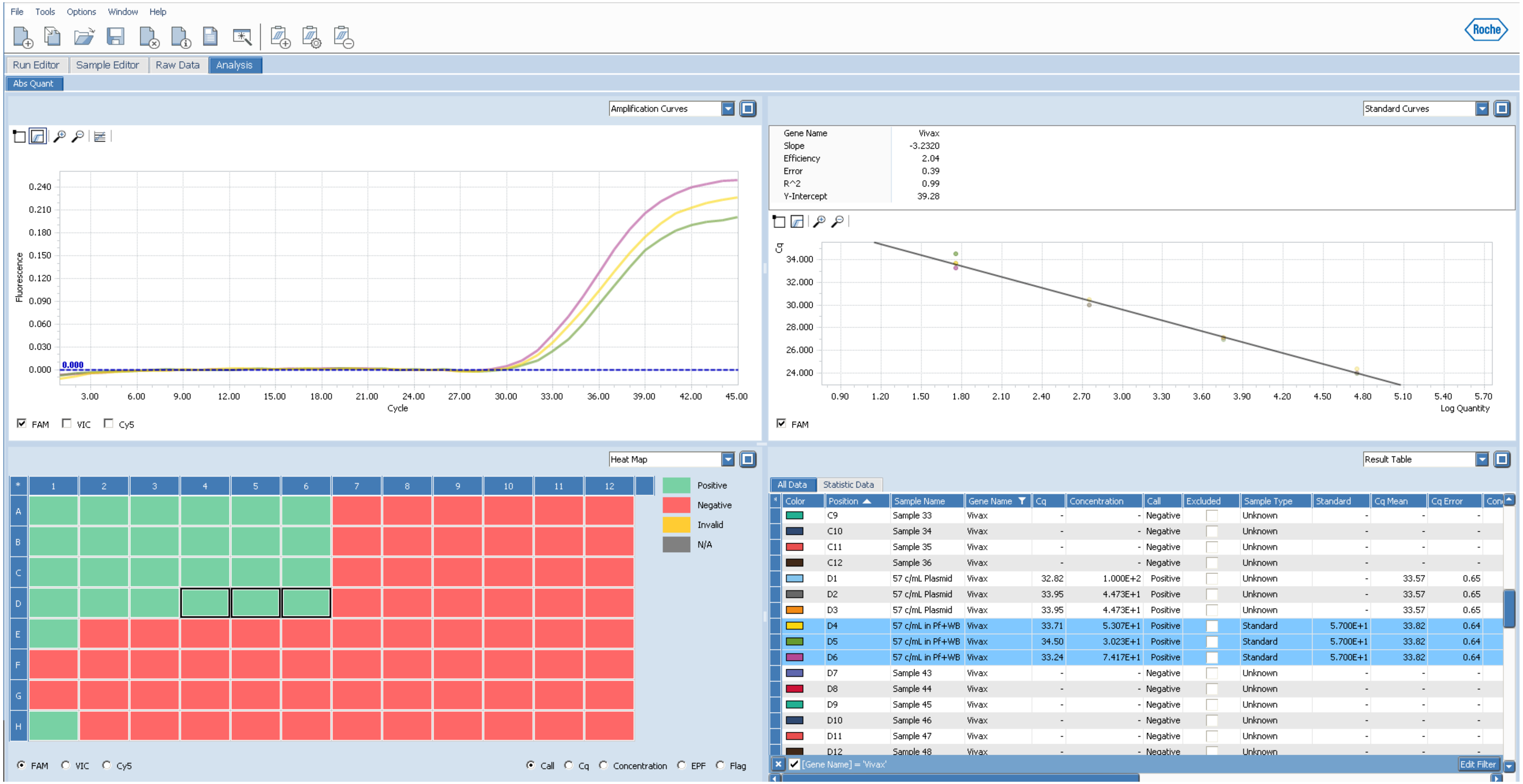

Supplementary Figure 3. Example Probit Graph of standard curve samples stored in tropical conditions for 3 days

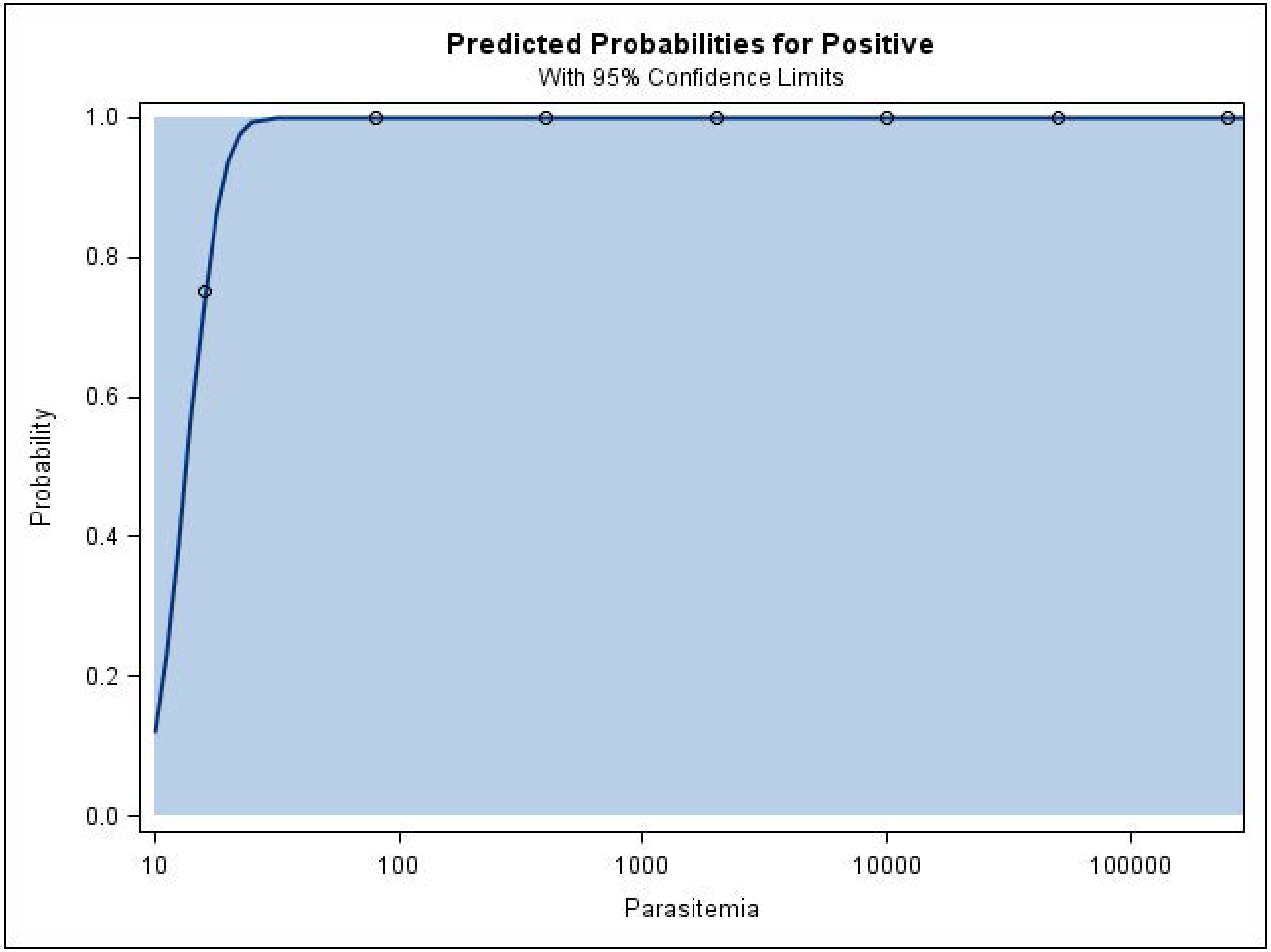

Supplement: Supplementary file 1 — 10.1186/s12936-015-1038-z Primers, probes, cycling condition. [file 12936_2015_1038_MOESM1_ESM.pdf]
